# The impact of chest pain center on treatment delay of STEMI patients: a time series study

**DOI:** 10.1186/s12873-021-00535-y

**Published:** 2021-11-06

**Authors:** Xiaolin Sun, Bo Yao, Kexin Shi, Yajiong Xue, Huigang Liang

**Affiliations:** 1 School of Management, Xi’an University of Finance and Economics, Xi’an, China; 2 School of Economics and Management, Xianyang Normal University, Xianyang, China; 3 Shaanxi Provincial People’s Hospital, Xi’an, China; 4 Center for Healthcare Management System, College of Business, East Carolina University, Greenville, NC 27858 USA; 5 Department of Business and Information Technology, Fogelman College of Business and Economics, University of Memphis, Memphis, TN 38152 USA

**Keywords:** ST segment elevation myocardial infarction, Chest pain center, Treatment delay

## Abstract

**Objective:**

To study the effect of the establishment of a Chest Pain Center (CPC) on the treatment delay of ST-elevation myocardial infarction (STEMI) patients and the influencing factors of treatment delay in a large hospital in China.

**Methods:**

The study subjects are 318 STEMI patients admitted between August 2016 and July 2019 to a large general hospital in Henan, China. Data were extracted from the electronic medical records after removing personal identifiable information. The interrupted time series regression was used to analyze the treatment delay of patients before and after the CPC establishment.

**Results:**

After the CPC establishment, the patients’ pre-hospital and in-hospital treatment delays were significantly reduced. SO-to-FMC (Symptom Onset to First Medical Contact time) decreased by 49.237 min and D-to-B (Door to Balloon time) decreased by 21.931 min immediately after the CPC establishment. In addition, SO-to-FMC delay is significantly correlated with age, occupation, nocturnal onset, and the way to hospital. D-to-B delay is significantly associated with time from initial diagnosis to informed consent of percutaneous coronary intervention (PCI), catheterization lab activation time, and time for PCI informed consent.

**Conclusion:**

The CPC significantly reduced the treatment delay of STEMI patients undergoing PCI.

**Supplementary Information:**

The online version contains supplementary material available at 10.1186/s12873-021-00535-y.

## Introduction

Acute Myocardial Infarction (AMI) is a serious coronary heart disease. With improved living conditions, faster pace of life, and stronger work pressures, people adopt unhealthy living habits and diets, and incidences of AMI are on the rise. As a type of ischemic heart disease (IHD), AMI has moved from the fourth place to the first place among all leading causes of early death from 1990 to 2017 in a global scope [1]. In China, IHD is the second leading cause of years of life lost in 2017 [2]. In the world, IHD is the second leading cause of disease burden as measured by disability adjusted life years [3]. AMI is a serious threat to human health and life. In 2017, the mortality rate of AMI among urban residents in China was 58.90 per ten thousand; in rural areas, the ratio is even higher, 76.04 per ten thousand [4].

To contain the harm of AMI, many countries have established a Chest Pain Center (CPC) to provide rapid diagnosis and treatment for patients with acute chest pain [5]. The center aims to shorten the time of diagnosis and treatment, reduce the pain of patients, curtail medical expenses, and improve the efficiency of treatment. By standardizing the diagnosis and treatment process and optimizing the diagnosis and treatment plan, CPCs can enhance quality of care and decrease the mortality rate of AMI [6]. Given the importance of chest pain centers in China, more research is needed to examine the clinical outcomes of CPCs, particularly the reduction of patients’ treatment time, before and after the CPC establishment.

AMI can be divided into STEMI (ST segment elevation myocardial infarction) and NSTEMI (non-ST segment elevated myocardial infarction), according to whether ST segment is elevated or not. According to the Chinese guidelines for the diagnosis and treatment of STEMI in 2019 [7], early, rapid and complete opening of the infarct related artery (IRA) is key to improve the prognosis of STEMI patients. The total time of myocardial ischemia, including patients’ own delay, pre-hospital system delay, and in-hospital treatment delay, should be shortened as far as possible. Given that STEMI has unique pathogenesis, clinical manifestations, and treatment methods, the mortality rate of STEMI patients in the short term is higher than other AMI patients. Timely percutaneous coronary intervention (PCI) can significantly improve the prognosis of STEMI patients [8]. Therefore, in this study, patients who suffered from STEMI and received PCI treatment were selected as the research subjects. We employed an interrupted time series model to investigate how myocardial ischemia time have changed before and after the CPC establishment.

## Methods

### Subjects

The research site is a grade III level A hospital in Henan, China. The hospital has 52 clinical departments, 1350 beds, and around 500,000 outpatients and 30,000 inpatients per year. The hospital established a CPC in April 2017 to optimize the treatment process of patients with chest pain. STEMI patients who were admitted to the emergency and outpatient department of the hospital during a three-year period (from August 2016 to July 2019) and received interventional operation were selected as the research subjects. A total of 318 patients were selected according to our inclusion and exclusion criteria.

### Inclusion and exclusion criteria

The diagnostic standards of the 2015 edition of the Guidelines for Acute STEMI Diagnosis and Treatment by the Chinese Society of Cardiology (CSC) and Guidelines for AMI Treatment (4th edition) by the European Society of Cardiology (ASC) in 2018 were taken as the inclusion criteria. Dynamic change patterns in electrocardiogram were used to diagnose STEMI patients. Only the patients who received confirmed STEMI diagnosis were included in this study. The exclusion criteria include: (1) severe liver and kidney dysfunction; (2) severe recent hemorrhagic diseases, such as cerebral hemorrhage, upper gastrointestinal hemorrhage; (3) severe hematological system diseases such as anemia; (4) combined conditions such as malignant tumors, rheumatic immune system diseases or serious infectious diseases; (5) undergoing coronary artery bypass grafting or valve replacement surgery. Patients with any of the conditions specified in the exclusion criteria were excluded from this study.

### Data sources

The patient data were retrieved from the CPC data platform, electronic medical records, and the HIS system of the hospital. The patient demographic characteristics include gender (0 = male, 1 = female), age, occupation (0 = farmers, 1 = company employees, 2 = government officials, 3 = retirees, 4 = migrant workers, 5 = unemployed, 6 = others), marital status (0 = single, 1 = married, 2 = divorced, 3 = widowed), and place of residence (0 = rural, 1 = urban). Patients’ current, previous and family medical history, and ways to be admitted were collected (0 = by self, 1 = by ambulance, 2 = inter-hospital transfer, 3 = intra-hospital transfer). We also collected clinical data such as patients’ state of consciousness, condition assessment, Killip classification, initial symptoms, main treatment time, and complications (including hypertension, diabetes, dyslipidemia, cerebral infarction). The initial typical symptom and atypical symptoms were recorded [9].

The clinical outcome measures include time of onset, time from symptom onset to first medical contact (SO-to-FMC), time from the first medical contact to the first electrocardiogram (FMC-to-ECG), bypass, catheter lab activation time, door-to-balloon (D-to-B) time, STEMI diagnosis to PCI, and the PCI compliance time. Time of onset is a binary variable indicating the time when STEMI took place (0 = nighttime, 1 = daytime). SO-to-FMC is measured as the interval (in minutes) between the time of onset and the time that medical staff come to help the patient. FMC-to-ECG is measured as the interval (in minutes) between the time of onset and the time that the first electrocardiogram is performed. Bypass is a binary variable that indicates whether the patient has bypassed the emergency department and critical care unit or not (0 = no bypass, 1 = bypass). Catheterization lab activation time is measured as the interval (in minutes) between the patient’s hospital arrival and the activation of the catheterization lab. D-to-B is measured as the interval (in minutes) between the patient’s arrival in the hospital and when a catheter guidewire crosses the culprit lesion in the cardiac catheter lab. STEMI diagnosis to PCI refers to the interval (in minutes) from the time that STEMI is diagnosed to the time that PCI is started. PCI compliance time refers to the time (in minutes) taken by the patient family to sign the informed consent to agree that PCI will be performed on the patient.

### Data analysis

Data was analyzed by using SPSS 19.0. The improvement of STEMI treatment time before and after the CPC establishment was analyzed by using Interrupted Time Series Analysis (ITS). ITS is a quasi-experimental research design, in which by collecting relevant data of measured results at multiple time points before and after the intervention and the intercepts and trends of the outcome indicators before and after the intervention are compared to evaluate the effect of the intervention on the outcome [10]. The two most common models of ITS are differential autoregressive integrated moving average model (ARIMA) and segmented regression analysis (SRA). We adopted SRA in this study.

In SRA, the time of implementation of the intervention is taken as an event. The entire study period is divided into 2 parts based on the event. A dummy variable is created to indicate before and after the intervention. Time points are used as independent variables, and regression analysis is performed on the time series data before and after the intervention. In SRA, the autocorrelation between the time trend and the observation result is considered simultaneously, and the time point of data collection is less required. Therefore, SRA is widely used in the study of intervention effects with relatively few data over time. The basic SRA model is:

$$ {Y}_t={\beta}_0+{\beta}_1 time+{\beta}_2 phase+{\beta}_3\mathrm{interact}+e $$

Before intervention:

$$ {Y}_t={\beta}_0+{\beta}_1 time+e $$

After intervention:

$$ {Y}_t={\beta}_0+{\beta}_1 time+{\beta}_2+{\beta}_3\mathrm{interact}+e=\left({\beta}_0+{\beta}_2\right)+\left({\beta}_1+{\beta}_3\right)\mathrm{time}+e $$

*Y*_*t*_ refers to the baseline value of the outcome at time *t*; *time* is the time point from the beginning of the study time and is a continuous variable; *phase* is a dummy variable: if the intervention implementation point is set to t', the value before the intervention is set to 0, and the value at and after the intervention is set to 1; *interact* is a continuous variable, the time point before intervention is taken as 0, and it is calculated according to t ‐ t '  + 1 from the intervention time point. *β*_*0*_ represents the estimated value of the outcome at baseline level, that is, at t = 0. *β*_*1*_ is the coefficient for time, indicating the slope of the regression curve before intervention. *β*_*2*_ is the coefficient for the intervention dummy, indicating the change of level at the time of intervention, that is, the difference between the end value of the outcome before intervention and the initial value of the outcome after intervention. *β*_*3*_ is the interaction coefficient, indicating the difference between the time trend after the intervention and the time trend before the intervention, that is, the change of the slope. e represents the random error term, which is a compensating error generated by various unobservable factors. Therefore, *β*_*2*_ represents the change in level, and *β*_*3*_ represents the change in slope before and after the intervention. Additional file 1: Appendix B shows a diagram to illustrate SRA.

The SO-to-FMC time of STEMI patients was used as an indicator of pre-hospital delay, and the D-to-B time was used as an indicator of in-hospital delay of the patients. The time-series data in the study was the monthly average of the time spent in each link of STEMI patients undergoing interventional operation. Using the CPC establishment as the discontinuity event, the time series was divided into 2 phases: before CPC phase (August 2016 to March 2017) and after CPC phase (April 2017–July 2019). Through the comparison of the time before and after the event, the CPC’s effect was analyzed.

In addition to ITS analyses, we identified the influencing factors of SO-to-FMC and D-to-B by employing binary logistic regressions. When *P* < 0.05, the effect was considered statistically significant.

## Results

### Patient demographics

Of the 318 patients, 273 were male and 45 female; their age was 57.78 ± 10.71; 129 had hypertension (76 in Stage 3, 34 in Stage 2, and 19 in Stage 1); 51 had type 2 diabetes; 22 had hyperlipidemia; 199 had smoking history (17 with smoking index ≤400, 182 with smoking index > 400); 107 had long-term excessive alcohol consumption (79 excessive alcohol consumption (25-59 g/d) and 28 heavy alcohol consumption (≥60 g/d)). Most of the 318 patients were farmers, accounting for 55.66%, followed by retirees (14.78%) and migrant workers (11.64%). Additional file 1: Appendix A shows the frequency distribution of different living habits and “three highs” (i.e., high blood pressure, glucose, and lipid) across genders in this study. Among them, hypertension accounted for 40.90%, type 2 diabetes accounted for 17.30%, dyslipidemia accounted for 6.9%, smoking accounted for 62.27%, and excessive alcohol consumption accounted for 33.64%.

Patients with STEMI have complex and varying clinical symptoms. In this study, the retrosternal pain was used as the initial typical symptom, and others were classified as the atypical symptoms. As shown in Additional file 1: Appendix A Table A2, 96.54% of the patients were found to show the typical symptom, accompanied by atypical symptoms after investigation and analysis of their medical records. For atypical symptoms, the most common were chest tightness and profuse sweating, followed by nausea, limb tiredness and weakness, vomiting, and radiating pain in the shoulder and back.

The patients took four different ways to be admitted to the CPC: coming on their own (137, 43.08%), calling an ambulance (48, 15.09%), transferring from another hospital (115, 36.16%), and getting sick in the hospital (18, 5.66%). The onset between 22:00 and 6:00 the next morning was defined as a nighttime onset, and the rest was the daytime onset. There were 257 cases (80.82%) of daytime onset and 61 cases (19.18%) night onset. Based on the assessment by the doctor when patients came to the hospital, the main manifestations were persistent or intermittent chest distress/pain, accounting for 79.87 and 15.72% of the total samples, respectively. According to the Killip classification, there were 294 (92.45%) patients in class I, 15 (4.72%) in class II, 7 (2.20%) in class III, and 2 (0.63%) in class IV.

Since the medical history, family history, and allergy history of the STEMI patients could influence the judgment of doctors, these data were collected, summarized, and analyzed. Among the 318 patients, 19 (5.97%) had a history of allergies, and 47 (14.78%) had siblings of parents or homologous parents who had heart disease. Meanwhile, 20 (12.58%) patients were having or formerly had cerebrovascular diseases, and 42 (13.21%) had AMI in the past.

### Changes in SO-to-FMC

The upper section of Table 1 shows the ITS analysis results of before-after changes of the SO-to-FMC time. The time-series equation before CPC is:

$$ {Y}_t=206.128-4.235 time+e, $$

and the equation after CPC is:

$$ {Y}_t=\left(206.128-85.078\right)+\left(3.978-4.235\right) time+e=121.05-0.27 time+e. $$

**Table 1:** Analysis results of changes in SO-to-FMC and D-to-B

Variables	*β*	Standard error	*t*	*p*	95%CI	
					Lower limit	Upper limit
DV: SO-to-FMC						
Constant term (β_0_)	206.128	19.434	10.607	0.000	166.327	245.929
Baseline trend (β_1_)	−4.235	3.83	−1.093	0.283	−12.079	3.609
Level change (β_2_)	−85.078	23.003	−3.699	0.001	− 132.188	− 37.968
Slope change (β_3_)	3.978	3.889	1.023	0.314	−4.135	11.943
DW	2.062					
R^2^	.566					
DV: D-to-B						
Constant term (β0)	184.202	12.067	15.265	0.000	159.489	208.915
Baseline trend (β1)	−9.241	2.405	−3.842	0.001	−14.166	−4.316
Level change (β2)	−103.642	14.487	−7.154	0.000	−133.311	−73.973
Slope change (β3)	9.079	2.420	3.751	0.001	4.123	14.035
DW	2.258					
R^2^	0.739					

The intercept of the jumping discontinuity is 49.237, and the slope change is 3.978. As shown in Fig. 2, in the before-CPC segment, SO-to-FMC showed a decreasing pattern, but the slope is not statistically significant, and the slope of the after-CPC segment is flattened, not statistically significant, either. Meanwhile there is a discontinuity jump at the time of CPC establishment, suggesting that the STEMI patients in the hospital had a significant decrease in the SO-to-FMC time right after the establishment of CPC.

**Fig. 1:**
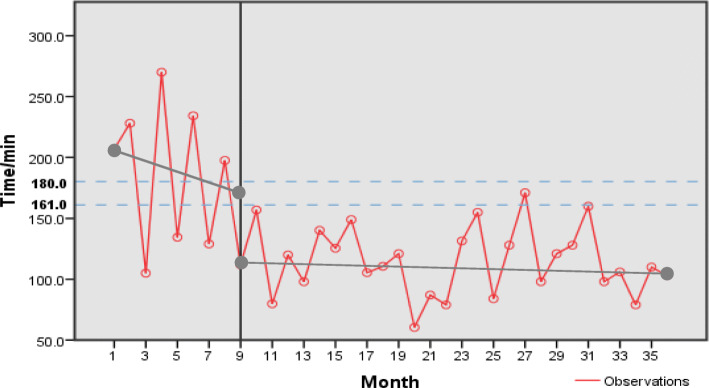
Time series chart of the SO-to-FMC time before and after CPC establishment

The results showed that the SO-to-FMC time after the construction of CPC was significantly reduced. In addition, the SO-to-FMC time after CPC was significantly lower than that required by the national standard (quarterly average time ≤ 180 min, the upper dashed line in Fig. 1) and the average time of all hospitals in China meeting the qualification in 2018 (quarterly average time was 161 min, the lower dashed line in Fig. 1). The results indicate that the SO-to-FMC time for STEMI patients has been improved after the CPC establishment, and the effect has been sustained over time.

### Changes in D-to-B time

The lower section of Table 1 shows the ITS analysis results of before-after changes of the D-to-B time. The time series equation before CPC is:

$$ {Y}_t=184.202-9.241 time+e, $$

and the equation after CPC is:

$$ {Y}_t=\left(184.202-103.642\right)+\left(9.079-9.241\right) time+e=80.56-0.162 time+e. $$

**Fig. 2:**
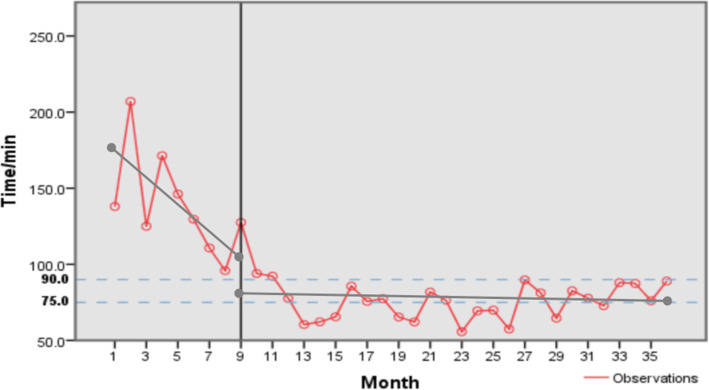
Time series chart of the SO-to-FMC time before and after CPC establishment

The analysis results showed that the D-to-B time in the hospital had improved significantly before and after the CPC establishment. The distance between jumping discontinuities was 21.931, the slope change was 9.079, and both are statistically significant. From Fig. 2, it could be clearly observed that the D-to-B time had changed from a clear downward trend to a flat line. This suggests that the D-to-B time was already declining before the CPC establishment; however, the CPC establishment still led to a sudden drop of D-to-B time. More important, the D-to-B time has consistently stayed at the low level since the CPC establishment. A plausible explanation for the before-CPC negative slope is that the hospital had started preparation for the CPC a few months earlier. During the preparation, the treatment process started to be standardized and optimized, which led to the decrease of D-to-B time. If we had more data further back, we could have seen a flatter line at a relatively high level.

In addition, the D-to-B time after CPC was significantly lower than that required by the national standard (quarterly average time ≤ 90 min, the upper dashed line in Fig. 2) and the average time of all hospitals in China meeting the qualification in 2018 (quarterly average time was 75 min, the lower dashed line in Fig. 2). The results indicate that the in-hospital time for STEMI patients has been improved after the CPC establishment, and the effect was sustainable over time.

### Impact factors of treatment delay

By analyzing the changes of patients’ treatment delay trends before and after the establishment of the CPC, we found that the treatment delay of STEMI patients in the hospital was significantly reduced. To explore why treatment was delays occurred, we further conducted logistic regression analyses to analyze the factors affecting the treatment delay of STEMI patients.

First, we analyzed what factors led to pre-hospital delay. Based on whether the treatment was delayed against the national standard (SO-to-FMC ≤ 180 min), the continuous variable of SO-to-FMC was converted into a binary variable (delayed = 1, not delayed = 0). This binary variable was the dependent variable, and the patient characteristics were used as independent variables. Nine indicators were included in logistic regression, including age, gender, marital status, occupation, have typical symptoms (yes = 1, no = 0), had an AMI before (yes = 1, no = 0), onset at night (yes = 1, no = 0), and ways of coming to hospital. Four factors were found to be significantly related to SO-to-FMC: age, occupation, ways to hospital, and nighttime onset.

As shown in Table 2, the OR of age was 1.049, suggesting that when age increases by 1, the chance that SO-to-FMC time is delayed will be increased by 4.9%. As to occupation, farmers were used as the control group because farmers were the largest group of the STEMI patients. As the results show, staff, civil servants, retirees, and workers had a lower risk of pre-hospital delay (OR < 1) than farmers, while patients without work and having other jobs had a higher risk of pre-hospital delay (OR > 1). Compared with patients coming to the hospital on their own, patients who were transferred, had an in-hospital onset, or called an ambulance had lower risk of pre-hospital delay (OR < 1). Finally, patients having a nocturnal onset had a much greater risk of pre-hospital delays than those having a daytime onset, increasing the risk by 109.6%.

**Table 2:** Logistic regression results for influencing factors of SO-to-FMC and D-to-B

Variables	*b*	*S.E*	*P*	*OR*
**DV: SO-to-FMC**				
Age	0.048	0.017	**0.005**	1.049
Occupation (reference: Farmers)			**0.033**	
Company employees	0.464	0.617	0.452	1.590
Government officials	−0.801	1.129	0.478	0.449
Retirees	−0.606	0.447	0.176	0.545
Other	2.166	1.183	0.067	8.727
None	−1.082	0.610	0.076	0.339
Migrant workers	− 1.707	0.781	0.029	0.181
Nocturnal onset (yes/no)	0.740	0.340	**0.030**	2.096
Way to hospital(reference: Self)			**0.005**	
Ambulance	−1.893	0.588	0.001	0.151
Inter-hospital transfer	−0.738	0.316	0.020	0.478
Intra-hospital transfer	−20.856	8932.347	0.998	0.00
**DV: D-to-B**				
Time of STEMI diagnosis to PCI (min)	0.017	0.004	**0.000**	1.017
Cath lab activation time (min)	0.168	0.078	**0.031**	1.183
Time for PCI informed consent (min)	0.651	0.286	**0.023**	1.917
Bypass ED/CCU (Yes = 1, no = 0)	−1.164	0.454	**0.010**	0.312

Second, we analyzed what factors led to in-hospital delay. Based on whether the in-hospital treatment was delayed against the national standard (D-to-B ≤ 90 min), the continuous variable of D-to-B was converted into a binary variable (delayed = 1, not delayed = 0). The binary D-to-B was the dependent variable, and four factors were included as independent variables for the logistical regression, including time of STEMI diagnosis to PCI, catheterization lab activation time, time for PCI informed consent, and whether the patient bypassed the emergency department and CCU. As the lower section of Table 2 shows, the first three factors can significantly increase the risk of D-to-B delay, particularly time for informed consent which increased the risk by 91.7%. Through interviews with some attending doctors, we found that when patients’ family members were not present to provide PCI consent, the patients’ condition was complicated, or the patients’ family members spent a long time in choosing the treatment method, delays in the D-to-B time are likely to occur. On the contrary, for patients who bypassed emergency department and coronary care unit, their D-to-B was 68.8% less likely to be delayed than those who did not bypass, suggesting that bypass is a critical factor for saving time and optimizing PCI treatment.

## Discussion

Through the pre-hospital and in-hospital emergency outpatient data, this study analyzed the improvement of STEMI patients’ treatment delay before and after the establishment of a chest pain center. The time series analysis results show that after the CPC establishment, the pre-hospital and in-hospital treatment delays were reduced. In order to explore the reasons for the delayed treatment of patients, logistic regressions were performed. The results revealed that age, occupation, night-time onset, and ways of visiting the hospital affected the pre-hospital delay. Regarding in-hospital delays, the time from the STEMI diagnosis to PCI and catheterization lab activation time reduced the risk of having D-to-B delay, and the time of signing PCI informed consent worsened the delay.

Our findings suggest the positive impact of CPCs in reducing STEMI patients’ treatment delay. For hospitals that have sufficient resources and capabilities, having a CPC could significantly optimize the treatment process and save precious time when treating STEMI patients. From the government’s perspective, support for chest pain centers should be strengthened. The development of CPCs in China face challenges in resource allocation and review process. In order to promote chest pain center, the government should increase investment in CPC resources, increase the number of reviewers, and streamline the review process.

Despite the importance of CPCs, many other factors can contribute to STEMI patients’ treatment delay [11]. One of the most important factors is public health education. Patients need to be educated to understand the criticality of time and seek help from CPCs whenever possible. After the onset of illness, patients may have difficulty in deciding whether to seek medical treatment in a timely manner and in what way to seek medical treatment. Patients’ health awareness needs to be improved to make wise decisions. It is urgent to strengthen health education of the public and urge them to pay attention to myocardial health. The concepts of “time is heart muscle” and “time is life” should be broadly disseminated in the communities to raise public awareness about the severity of myocardial infarction and the importance of prevention and treatment. It is important to popularize the causes of AMI, improve self-care awareness, and improve self-help and rescue skills in emergencies. Particularly, rural areas are the hardest hit by AMI in China. It is critical to educate rural people to grasp the correct first aid methods to seek medical help as early as possible in case of AMI onset.

This study has two limitations. First, there was no control group. Although we found a significant decline in SO-to-FMC and D-to-B time, it should be noted that the study is observational and cannot reflect the causal effect of the chest pain center. A natural experiment with a control group is recommended for future research to confirm our findings. Second, this study was conducted in a single hospital. The unique characteristics of the hospital context and the way its CPC was implemented could influence the treatment delays of STEMI patients. Future studies should collect data from multiple hospitals to mitigate this concern.

## Conclusion

Based on an observational study of 318 STEMI patients over 3 years, we find that the SO-to-FMC and D-to-B time was significantly shortened after the establishment of a CPC. We also identified several factors that could influence SO-to-FMC and D-to-B time, respectively.

## Supplementary Information

**Additional file 1: Appendix A.** Sample characteristics. **Table A1.** STEMI patient characteristics. **Table A2.** Frequency of clinical symptoms among STEMI patients. **Appendix B.** Illustration of segmented regression analysis. **Figure B1.** Analysis logic of SRA.

## Data Availability

The datasets collected and analyzed during the current study are available based on reasonable request by sending emails to: sunxiaolin706@163.com.
