# Supplementary material for: The impact of chest pain center on treatment delay of STEMI patients: a time series study
Source: BMC Emerg Med. 2021 Nov 6;21:129. doi: 10.1186/s12873-021-00535-y (PMC8571845; doi:10.1186/s12873-021-00535-y)
Supplement: Supplementary file 1 — Additional file 1: Appendix A. Sample characteristics. Table A1. STEMI patient characteristics. Table A2. Frequency of clinical symptoms among STEMI patients. Appendix B. Illustration of segmented regression analysis. Figure B1. Analysis logic of SRA. [file 12873_2021_535_MOESM1_ESM.docx]

**Appendix A: Sample characteristics**

Table A1. STEMI patient characteristics

| variables | group | Male | female | total |
| --- | --- | --- | --- | --- |
| Hypertension | None | 166（52.20%） | 22（6.90%） | 188（59.10%） |
|  | Level 1 | 17（5.40%） | 3（0.90%） | 20（6.30%） |
|  | Level 2 | 29（9.10%） | 5（1.60%） | 34(10.70%) |
|  | Level 3 | 61（19.20%） | 15(4.70%) | 76(23.90%) |
| Type2 diabetes | None | 234（73.60%） | 29（9.10%） | 263（82.70%） |
|  | Yes | 39（12.30%） | 16（5.00%） | 55（17.30%） |
| Dyslipidemia | None | 256（80.50%） | 40（12.60%） | 296(93.10%) |
|  | Yes | 17(5.30%) | 5(1.60%) | 22(6.90%) |
| Smoke index | 0 | 75(23.58%) | 45(14.15%) | 120(37.73%) |
|  | ≤400 | 17(5.35%) | 0(0.00%) | 17(5.35%) |
|  | >400 | 181(56.92%) | 0(0.00%) | 181(56.92%) |
| Excessive drinking | Never drinking/Moderate drinking | 166(52.20%) | 45(14.15%) | 211(66.36%) |
|  | Excessive drinking | 79(24.84%) | 0(0.00%) | 79(24.84%) |
|  | Heavy drinking | 28(8.81%) | 0(0.00%) | 28(8.81%) |

Table A2. Frequency of clinical symptoms among STEMI patients

| variables | Yes | No |
| --- | --- | --- |
| Pain behind the sternum | 307（96.54%） | 11（3.46%） |
| Chest tightness | 281（88.36%） | 37（11.64%） |
| Profuse sweating | 246（77.36%） | 72（22.64%） |
| Nausea | 111（34.91%） | 207（65.09%） |
| Limbs aching and limp | 71（22.33%） | 241（77.67%） |
| Vomit | 63（19.81%） | 255（80.19%） |
| Shoulder pain | 60（18.87%） | 258（81.13%） |
| Dizziness | 34（10.69%） | 284（89.31%） |
| Throat uneasiness | 28（8.81%） | 290（91.19） |
| Numb | 10（3.14%） | 308（96.86%） |
| Amaurosis | 9（2.83%） | 309（97.17%） |
| Convulsion | 3（0.94%） | 315（99.06%） |
| Incontinence | 2（0.63%） | 316（99.37%） |

**Appendix B. Illustration of segmented regression analysis**


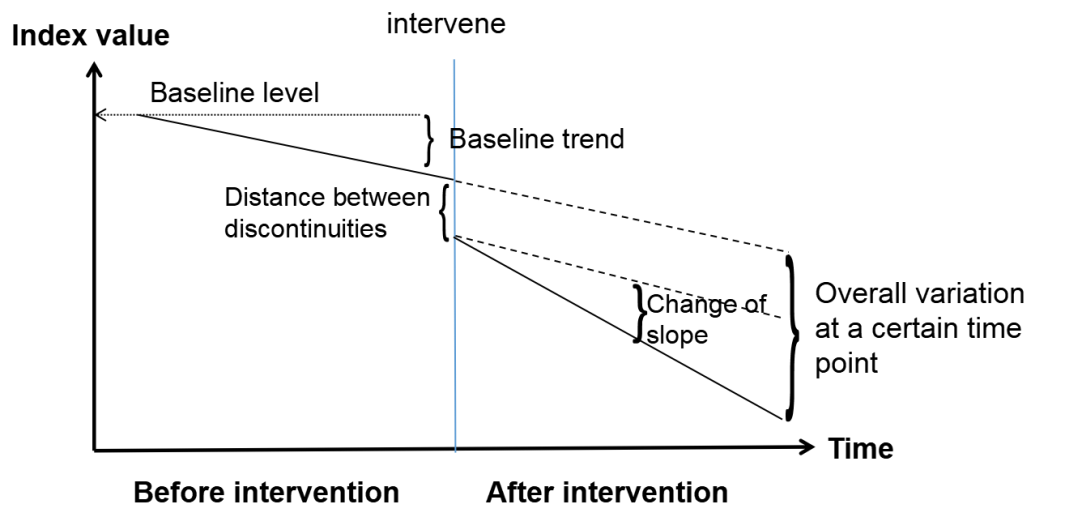


Figure B1. Analysis logic of SRA
